# Effects of five-minute internet-based cognitive behavioral therapy and simplified emotion-focused mindfulness on depressive symptoms: a randomized controlled trial

**DOI:** 10.1186/s12888-017-1248-8

**Published:** 2017-03-04

**Authors:** Remi Noguchi, Yoichi Sekizawa, Mirai So, Sosei Yamaguchi, Eiji Shimizu

**Affiliations:** 1 Department of Cognitive Behavioral Physiology, Graduate School of Medicine, Chiba University, 1-8-1 Inohana, Chuo-ku, Chiba, 260–8670 Japan; 2 Research Institute of Economy, Trade and Industry, 1-3-1 Kasumigaseki, Chiyoda-ku, Tokyo, 100-8901 Japan; 3 Department of Neuropsychiatry, Keio University School of Medicine, 35 Shinanomachi, Shinjuku-ku, Tokyo, 160-8582 Japan; 4 Department of Psychiatric Rehabilitation, National Institute of Mental Health, National Center of Neurology and Psychiatry (NCNP), 4-1-1 Ogawa-Higashi, Kodaira, Tokyo, 187-8553 Japan; 5 Cognitive Behavioral Therapy Center, Research Center for Child Mental Development, Department of Cognitive Behavioral Physiology, Graduate School of Medicine, Chiba University, 1-8-1 Inohana, Chuo-ku, Chiba, 260–8670 Japan

**Keywords:** Depression, Five-minute internet-based cognitive behavioral therapy, Simplified emotion-focused mindfulness, Emotional acceptance

## Abstract

**Background:**

Notwithstanding a high expectation for internet-based cognitive behavioral therapy (iCBT) for reducing depressive symptoms, many of iCBT programs have limitations such as temporary effects and high drop-out rates, possibly due to their complexity. We examined the effects of a free, simplified, 5-minute iCBT program by comparing it with a simplified emotion-focused mindfulness (sEFM) exercise and with a waiting list control group.

**Methods:**

A total of 974 participants, who were recruited using the website of a market research company, were randomly assigned to the iCBT group, the sEFM group, and the control group. Those in the intervention arms performed each exercise for 5 weeks. The primary outcome measure was the Center for Epidemiological Studies Depression scale (CES-D) at postintervention. Secondary outcome measures were the Patient Health Questionnaire-9 (PHQ-9) and the Generalized Anxiety Disorder-7 scale (GAD-7). Intention-to-treat analyses were conducted.

**Results:**

During postintervention assessment, there were no significant differences between the intervention arms and the control group in the CES-D, although the difference between the iCBT arm and control group was close to significance (*p* = 0.05) in favor of iCBT. There was a significant difference in the PHQ-9 in favor of the sEFM group compared with the control group. There were no significant differences in outcome measures between the three groups at the 6-week follow-up.

**Conclusions:**

Although both iCBT and sEFM have the potential to temporarily reduce depressive symptoms, substantial improvements are required to enhance and maintain their effects.

**Trial registration:**

This trial is registered with the UMIN Clinical Trial Registry (UMIN-CTR) (ID: UMIN000015097) on 1 October 2014.

**Electronic supplementary material:**

The online version of this article (doi:10.1186/s12888-017-1248-8) contains supplementary material, which is available to authorized users.

## Background

Depression is a common mental health problem. Approximately 350 million people worldwide are suffering from depression. Considering the importance of dealing with depression, the United States Preventive Services Task Force recommended the initiation of a general screening of depression for the adult population [1]. From December 2015, Japan went to the extent of obliging companies with 50 or more employees to perform stress checks, which are an annual examination of employees’ stress levels, including symptoms of depression and anxiety [2]. Such a trend for general checks for depression may result in the more frequent identification of people suffering from depression and more people seeking treatment for it.

The likely increase in detection of depressed people through a general screening should be followed by systematic and effective treatments. While antidepressants are the first-line treatment of depression in many countries, there is a growing interest in the use of psychotherapy, especially cognitive behavioral therapy (CBT). However, the number of adequately trained CBT therapists is extremely low in many countries, including Japan. Thus, the development of other delivery methods for CBT is necessary for the effective prevention and treatment of depression. To deal with this problem, internet-based CBT (iCBT) has gained attention and numerous studies have analyzed the effects of iCBT on depression. Although the potential effects of iCBT in reducing depressive symptoms have been shown in meta-analyses [3, 4], the effects were observed to be transient and the participants’ drop-out rate was high [5].

We approached this problem by focusing on two modalities—simplified 5-minute iCBT and simplified emotion-focused mindfulness (sEFM)—and comparing them with each other and with a waiting list control group. The 5-minute iCBT exercise was a simple iCBT that asked the participants to identify stressful thoughts, come up with thoughts opposite to the original ones, and then look for evidence to support the opposite thoughts. The sEFM exercise in the study involved practicing the acceptance of negative feelings and, if a person did not feel negative feelings at the moment, coming up with recent events invoking a small measure of negative emotion. The idea behind using our online intervention for depression in the present study is simplicity. Most of the pre-existing iCBT programs comprise several steps and many things to learn and practice and can thus be somewhat complicated. This complexity may lead to a high drop-out rate among participants. In addition, some iCBT programs are not free and users must pay for them without any assurance that they will work. In the current study, we examined the effect of a simple and free online exercise for depression.

The sEFM exercise in the present study is the practice of a nonjudgmental attitude toward negative emotions. Mindfulness is nonjudgmental attention paid to moment-to-moment experiences [6]. Several therapeutic modalities, such as acceptance and commitment therapy [7] and mindfulness-based cognitive therapy [8], emphasize the importance of a nonjudgmental attitude, including acceptance. Although other mindfulness exercises tend to focus on the awareness of breath and bodily sensations [6], a sEFM exercise focuses only on emotions. Hence, this exercise accommodates the concept of emotional acceptance as in the context of emotion regulation [9]. Several studies on emotion regulation [10] have shown that a strategy of emotional acceptance is effective in reducing negative emotions such as sadness [11–14] and increasing positive affect [15] in short-term experiments. However, the long-term effects of emotional acceptance are unclear [16, 17].

Comparing the effects of simplified iCBT and sEFM is meaningful in the following ways. First, another type of effective self-help interventions for depression may be available to those in need. Second, a comparison of rather long-term effects of cognitive reappraisal and emotional acceptance may be possible. A key component of the iCBT exercise in the present study is cognitive restructuring, in which a person re-examines the interpretation or meaning of a negative stimulus by coming up with evidence to the contrary. Cognitive restructuring is considered as a type of cognitive reappraisal, which involves modifying the meaning of a stimulus or context that gives rise to an emotion [18]. Several studies have carried out comparisons of cognitive reappraisal and emotional acceptance [11, 12, 19]. However, to the authors’ knowledge, there are no studies that compare these two forms of emotion regulation in a real-world setting and that follow a duration longer than a few months.

Therefore, the objective of the present study is to compare the effects of simplified iCBT and sEFM exercises on depressive symptoms with each other and also with a waiting list control group through a web-based, large-scale randomized controlled trial (RCT).

## Methods

### Design

The present study is a pragmatic RCT with a 1:1:1 allocation into three arms: a simplified iCBT, a sEFM, and a waiting list control group. The assessment points were at baseline (T0), postintervention (T1), six weeks after T1 (T2), and 6 weeks after T2 (T3). Those in the waiting list control group received either the iCBT or the sEFM intervention based on randomization just after assessment at T2. Thus, there were no comparisons between the intervention groups and the waiting list group at T3. The study was approved by the Ethics Review Committee of the Chiba University Graduate School of Medicine and registered with the UMIN Clinical Trial Registry (UMIN-CTR) (ID: UMIN000015097).

### Participants and recruitment

The participants in the present study were recruited by a Japan-based market research company from May 28 to June 2, 2015. On the basis of the contract between the company and the Research Institute of Economy, Trade and Industry (RIETI), to which one of the authors (Y. S.) belonged, the company sent an invitation e-mail to people who had registered on its website to take part in surveys conducted by the company. In the invitation e-mail, the research theme of examining the effect of a mental health promotion exercise was announced and recipients of the e-mail were invited to log in to the website showing the details of the research. Individuals who accessed the website were asked to answer the initial screening (T00) questions, including questions from the Center for Epidemiological Studies Depression scale (CES-D) [20], Patient Health Questionnaire-9 (PHQ-9) [21], and other questions to confirm whether they met other inclusion criteria.

The inclusion criteria were (i) showing symptoms of at least mild depression (CES-D ≥ 16 and PHQ-9 ≥ 5), (ii) being older than 19 years of age and younger than 66 years of age at the time of the recruitment, (iii) having no suicidal ideation (a score of 0 or 1 on item 9 on the PHQ-9), (iv) having internet access, (v) having time to do the exercise for 5 to 10 min twice per week for 5 weeks, (vi) being interested in doing the program, and (vii) being willing to help with the research to be conducted.

Those who met the inclusion criteria were shown a detailed and printable explanation of the study on the website, and only those who gave informed consent online proceeded to the baseline assessment (T0). There was a week-long interval between initial screening (T00) and baseline assessment (T0). Participants were included in the study if their CES-D and PHQ-9 scores met inclusion criteria at the initial screening (T00), even if they no longer scored above this threshold at the baseline assessment (T0). This is because it seemed impractical, offensive, and rather unethical to deny an individual participation in the study after he or she had given informed consent and a required baseline assessment (T0).

### Randomization and masking

Those who responded to the baseline assessment were randomly assigned to the three groups. Randomization sequence was created using Stata 12.1 (StataCorp, College Station, TX) with a 1:1:1 allocation using random block sizes of 3, 6, 9, 12, and 15. An independent researcher conducted the block randomization.

Given the nature of the intervention, the participants were not masked regarding which intervention they were engaged in. As all outcome measures were collected through an automated online procedure, the masking of outcome assessors was not necessary.

### Procedures

The participants assigned to the iCBT or sEFM groups were sent e-mails every day encouraging them to log in to the website created for the exercise. The e-mails also included tips for doing the exercise. The content of the tips changed every week. A Frequently Asked Questions resource for the exercise was available for the participants on the website of the market research company. By logging in to the website, each participant was able to perform the exercise. The exercise continued for five weeks. Although whether or not the participants accessed the website for each exercise could not be determined, the participants’ responses to the exercise were sent electronically to the market research company. Therefore, authors were able to verify whether the participants actually performed their allotted exercises. The participants’ responses to the exercise were kept confidential. This confidentiality was conveyed to the participants prior to the study. There was no contact between the researchers and participants except the answers to the questions received from the participants via e-mail. The participants were informed that they would receive remuneration based on the following conditions. Those who performed the exercise twice per week for the entire 5 weeks received 1000 yen (approximately $10). They also received 500 yen as they responded to each of the assessment questionnaires from T1 to T3. One hundred people who did not perform the exercise twice per week for the entire five weeks but answered either of the assessment questionnaires at each point from T1 to T3 received 500 yen through a lottery.

### Interventions

The simplified iCBT program used in the study was developed by one of the authors (E. S.). This is a straightforward program that identifies stress-generating cognitions (thoughts) and encourages participants to come up with the opposite thoughts and find evidence and examples to support the new thoughts, which in turn encourages them to make a cognitive restructuring. Instructions for the exercise are shown in Additional file 1. The participants were able to fill in their answer on the website.

sEFM is a simple mindfulness exercise in which participants are instructed to take time to feel their negative emotions without judgment. In previous studies in which participants were encouraged to accept their feelings, they were induced to feel negative emotions by, for example, watching horror movies [12] or writing about negative events [11]. Because we were unable to use such short-term inducements in the present study, we instructed the participants to come up with recent experiences that were slightly uncomfortable. Instructions for the exercise are shown in Additional file 2. The participants were instructed to fill in their comments on the website after each session of the exercise.

### Outcomes

All of the outcome measures in the present study were collected using a web-based self-report questionnaire at T0, T1, T2, and T3.

The primary outcome measure was the CES-D at T1. The CES-D is a 20-item scale that assesses the severity of depressive symptoms experienced during the previous week [20]. The Japanese translation of the CES-D was used in the present study [22]. CES-D scores ranged from 0 to 60, with higher scores indicating a higher level of depression.

Secondary outcome measures included the PHQ-9 [21], which is a 9-item scale that assesses the severity of depressive symptoms experienced during the past 2 weeks. PHQ-9 scores range from 0 to 27, with higher scores indicating higher levels of depression. The Japanese translation of the PHQ-9 was used [23]. We also recorded anxiety symptoms using the Generalized Anxiety Disorder-7 scale (GAD-7) [24], which is a 7-item scale that assesses the symptoms of general anxiety disorder. GAD-7 scores range from 0 to 21, with higher scores indicating a higher level of anxiety. The Japanese translation of the GAD-7 was used [25].

### Statistical analysis

To the best of our knowledge, there have been no RCTs comparing iCBT and sEFM. However, in a similar study, an RCT compared internet-delivered interpersonal psychotherapy with iCBT, and its between-group effect size was 0.09 [26]. To detect an effect size of 0.1, a power calculation with 80% power and an α-error probability of 0.05 resulted in a total sample size of 967 participants. This power calculation was conducted using the G*power Version 3.1.9.2 [27, 28].

Linear mixed-effects models for repeated measures were conducted using a group (iCBT, sEFM, or waiting list) × time (T0, T1, T2) interaction as an indicator of intervention effect. A random intercepts model was run using the restricted maximum likelihood estimation procedure and an unstructured covariance matrix. A major merit of using an unstructured matrix is that no restrictions are placed on the variances and covariances, and this choice can be attractive in studies such as the present one, in which the number of measurement timings are few [29]. Covariates were each outcome measures at baseline. All statistical analyses were conducted using STATA12, 13, and 14. The significance level was set at 5% (two-tailed). Intention-to-treat analyses were conducted.

As there were participants whose baseline depression level was below the inclusion criteria (CES-D ≥ 16 and PHQ-9 ≥ 5) because of the lag between initial screening (T00) and baseline assessment (T0), subgroup analyses limiting to those who met these criteria at both screening (T00) and baseline assessment (T0) were also conducted. Subgroup analyses were also conducted depending on the severity of depression at baseline. For the CES-D, level of depression was defined as follows: nondepression as CES-D < 16, mild depression as 16 ≤ CES-D < 26, and moderate and more severe depression as CES-D ≥ 26 [30]. For the PHQ-9, level of depression was defined as follows: nondepression as PHQ-9 < 5, mild depression as 5 ≤ PHQ-9 < 10, and moderate and more severe depression as PHQ-9 ≥ 10 [21].

## Results

### Participants

**Fig. 1:**
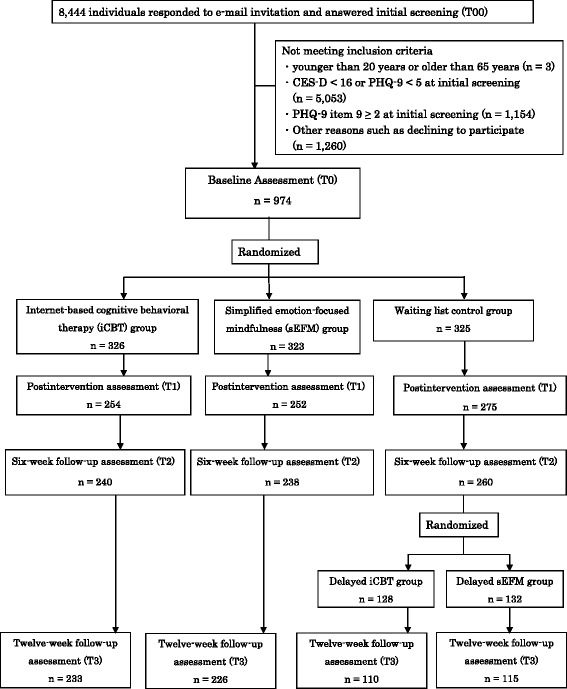
Participant flowchart

A flow chart of the participants is shown in Fig. 1. A total of 8444 people responded to the invitation e-mail. After excluding those who did not meet the inclusion criteria, a total of 974 people gave informed consent and completed a baseline survey. The participants were divided into the following groups: 326 people in the iCBT arm, 323 in the sEFM arm, and 325 in the waiting list arm. The demographic characteristics of the participants at baseline are shown in Table 1. There were no significant differences between the three groups in terms of sex, age, marital status, educational attainment, employment status, CES-D score at T0, PHQ-9 score at T0, and GAD-7 score at T0.

**Table 1:** Demographic characteristics of participants at baseline

		iCBT	sEFM	Waiting list	Total
Sex	Male	168 (51.5%)	159 (49.2%)	159 (48.9%)	486 (49.9%)
	Female	158 (48.5%)	164 (50.8%)	166 (51.1%)	488 (50.1%)
Age	Years (SD)	44.3 (11.3)	43.3 (11.3)	43.4 (11.3)	43.7 (11.3)
Marriage					
	Married	169 (51.8%)	170 (52.6%)	186 (57.2%)	525 (53.9%)
	Divorced	29 (8.9%)	23 (7.1%)	21 (6.5%)	73 (7.5%)
	Widowed	1 (0.3%)	1 (0.3%)	1 (0.3%)	3 (0.3%)
	Never married	127 (39.0%)	129 (39.9%)	117 (36.0%)	373 (38.3%)
Highest education level					
	Junior high school	5 (1.5%)	1 (0.3%)	1 (0.3%)	7 (0.7%)
	Senior high school	59 (18.1%)	66 (20.4%)	79 (24.3%)	204 (20.9%)
	Two-year college	57 (17.5%)	69 (21.4%)	61 (18.8%)	187 (19.2%)
	Four-year college or more	205 (62.9%)	187 (57.9%)	184 (56.6%)	576 (59.1%)
Employment Status					
	Employed	237 (72.7%)	240 (74.3%)	234 (72.0%)	711 (73.0%)
	Not employed and seeking job	23 (7.1%)	23 (7.1%)	22 (6.8%)	68 (7.0%)
	Not employed	66 (20.2%)	60 (18.6%)	69 (21.2%)	195 (20.0%)

*iCBT* internet-based cognitive behavioral therapy, *sEFM* simplified emotion-focused mindfulness

Of the participants who were assigned to the iCBT or sEFM arms, 254 out of 326 (77.9%) in the iCBT arm and 252 out of 323 (78.0%) in the sEFM arm completed the postintervention assessment at T1. In the waiting list arm, 275 of 325 participants (84.6%) completed the assessment at T1.

### Outcome measures including T00

The means and standard deviations for outcome measures on all responses are shown in Table 2. Scores of CES-D and PHQ-9 at initial screening for the participants were also shown (T00). Paired t-tests showed that there were no significant differences in CES-D and PHQ-9 between T00 and T0 (CES-D: *t* = 0.70, *p* = 0.49 and PHQ-9: *t* = 1.17, *p* = 0.24), whereas both significantly decreased from T0 to T1 (CES-D: *t* = 3.97, *p* < 0.001 and PHQ-9: t = 3.71, *p* < 0.001).

**Table 2:** Means and standard deviations at each time point (all responses)

		iCBT			sEFM			Waiting list		
	Time	N	M	(SD)	N	M	(SD)	N	M	(SD)
CES-D	T00	326	24.76	(7.70)	323	24.82	(7.48)	325	24.15	(6.79)
	T0	326	24.87	(9.15)	323	24.41	(8.57)	325	23.99	(8.06)
	T1	254	23.58	(9.62)	252	23.42	(10.00)	275	23.38	(9.17)
	T2	240	24.08	(10.02)	238	23.98	(10.02)	260	23.00	(9.22)
	T3	233	22.98	(10.18)	226	23.61	(10.18)	225	21.16	(9.26)
PHQ-9	T00	326	9.88	(3.75)	323	9.69	(3.93)	325	9.88	(3.80)
	T0	326	9.75	(5.06)	323	9.61	(5.06)	325	9.66	(4.54)
	T1	254	9.30	(4.87)	252	8.69	(5.52)	275	9.47	(5.14)
	T2	240	9.87	(5.49)	238	9.24	(5.63)	260	9.27	(5.20)
	T3	233	9.31	(5.64)	226	9.31	(5.85)	225	8.32	(4.65)
GAD-7	T0	326	7.10	(4.63)	323	7.09	(4.88)	325	6.87	(4.29)
	T1	254	6.66	(4.70)	252	6.65	(4.85)	275	6.60	(4.53)
	T2	240	7.26	(5.17)	238	7.05	(5.27)	260	6.61	(4.62)
	T3	233	6.70	(4.99)	226	7.19	(5.16)	225	6.30	(4.52)

*iCBT* internet-based cognitive behavioral therapy, *sEFM* simplified emotion-focused mindfulness. T00 = initial screening (only for CES-D and PHQ-9), T0 = baseline, T1 = postintervention, T2 = six weeks after T1, T3 = six weeks after T2. *CES-D* the Center for Epidemiological Studies Depression scale, *PHQ-9* the Patient Health Questionnaire-9, *GAD-7* the Generalized Anxiety Disorder-7

### Effects of the iCBT and sEFM compared with those of the waiting list

#### Analysis of primary outcome

We compared each intervention group with the waiting list group. The results are shown in Table 3. Regarding the CES-D, which is the primary outcome measure, the intervention effects estimated by the mixed-effects model analysis at T1 (postintervention) were not significant, although the CES-D score at T1 of the iCBT arm was lower with marginal significance as compared with that at T1 of the waiting list arm (−1.28, 95% CI: −2.58 to 0.02, *p* = 0.05).

**Table 3:** Linear mixed model analyses

		Predicted means (95% CI)			Intervention effect (95% CI), *p* value		
	Time	iCBT	sEFM	Waiting list	iCBT – waiting list	sEFM – waiting list	iCBT – sEFM
CES-D	T0	24.66 (23.98, 25.35)	24.56 (23.88, 25.25)	24.47 (23.79, 25.16)			
	T1	22.87 (22.10, 23.64)	23.34 (22.56, 24.11)	23.96 (23.22, 24.71)	−1.28 (−2.58, 0.02); 0.05	−0.72 (−2.02, 0.59); 0.28	−0.56 (−1.88, 0.76); 0.41
	T2	23.14 (22.35, 23.94)	23.88 (23.08, 24.67)	23.49 (22.73, 24.25)	−0.53 (−1.86, 0.80); 0.43	0.30 (−1.03, 1.63); 0.66	–0.83 (−2.18, 0.52); 0.23
	T3	22.07 (21.22, 22.93)	23.52 (22.65, 24.38)				**−1.56 (−2.94, –0.18); 0.03**
PHQ-9	T0	9.74 (9.36, 10.11)	9.71 (9.33, 10.09)	9.72 (9.34, 10.10)			
	T1	9.00 (8.57, 9.42)	8.68 (8.25, 9.11)	9.66 (9.25, 10.07)	−0.68 (−1.42, 0.05); 0.07	**−0.97 (−1.70, −0.23); 0.01**	0.29 (−0.46, 1.03); 0.45
	T2	9.52 (9.08, 9.96)	9.25 (8.81, 9.69)	9.50 (9.08, 9.92)	0.00 (−0.74, 0.75); 0.99	−0.24 (−0.99, 0.51); 0.53	0.24 (−0.52, 1.00); 0.53
	T3	8.94 (8.47, 9.41)	9.24 (8.76, 9.72)				−0.34 (−1.12, 0.44); 0.40
GAD-7	T0	7.09 (6.75, 7.43)	7.09 (6.75, 7.42)	7.04 (6.70, 7.38)			
	T1	6.38 (6.00, 6.77)	6.54 (6.16, 6.93)	6.79 (6.42, 7.15)	−0.45 (−1.10, 0.20); 0.18	−0.29 (–0.94, 0.37); 0.39	−0.16 (−0.83, 0.50); 0.63
	T2	7.01 (6.62, 7.40)	7.03 (6.64, 7.42)	6.84 (6.46, 7.22)	0.12 (−0.54, 0.79); 0.72	0.14 (−0.53, 0.81); 0.68	−0.02 (−0.69, 0.66); 0.96
	T3	6.45 (6.03, 6.87)	7.17 (6.75, 7.59)				**−0.72 (−1.41, −0.04); 0.04**

*iCBT* internet-based cognitive behavioral therapy, *sEFM* simplified emotion-focused mindfulness. *CI* confidence interval. T0 = baseline, T1 = postintervention, T2 = six weeks after T1, T3 = six weeks after T2. *CES-D* the Center for Epidemiological Studies Depression scale, *PHQ-9* the Patient Health Questionnaire-9, *GAD-7* the Generalized Anxiety Disorder-7. For T0 through T2, predicted means (95% CI) from mixed model with outcome measure at baseline, time (T0, T1, T2), treatment (iCBT, sEFM, Waiting list), and interaction between time and treatment as fixed effects. For T3, predicted means (95% CI) from mixed model with outcome measure at baseline, time (T0, T1, T2, T3), treatment (iCBT, sEFM, delayed iCBT, delayed sEFM), and interaction between time and treatment as fixed effects. Fixed effect of time × treatment interaction as an indicator of intervention effect. Values in bold are significant at 5%

#### Analysis of secondary outcomes

The results are shown in Table 3. Regarding PHQ-9 as the secondary outcome, there was a non-significant difference in favor of the iCBT (−0.68, 95% CI: −1.42 to 0.05, *p* = 0.07) and a significant difference in favor of the sEFM arm (−0.97, 95% CI: −1.70 to −0.23, *p* = 0.01) as compared with the waiting list arm at T1. These differences were not maintained at T2 (six weeks after T1).

There were no significant differences on the GAD-7 between any group and at any time point.

### Comparison of iCBT and sEFM

The results are shown in Table 3. Comparisons of the iCBT arm and the sEFM arm showed that there were significant differences at T3 (six weeks after T2) in favor of iCBT on the CES-D and GAD-7 (−1.56, 95% CI: −2.94 to −0.18, *p* = 0.03; −0.72, 95% CI: −1.41 to −0.04, *p* = 0.04, respectively).

### Analyses of subgroup that met the inclusion criteria at both screening (T00) and baseline assessment (T0)

The means and standard deviations for outcome measures on all responses for the participants limiting to those who met the inclusion criteria at both screening (T00) and baseline assessment (T0) (CES-D ≥ 16 and PHQ-9 ≥ 5) are shown in Table 4. Those were 264 people in the iCBT arm, 249 in the sEFM arm, and 266 in the waiting list arm. The results of those who met the inclusion criteria at baseline are shown in Table 5.

**Table 4:** Means and standard deviations at each time point (those who met inclusion criteria at baseline (CES-D ≥ 16 and PHQ-9 ≥ 5))

		iCBT			sEFM			Waiting list		
	Time	N	M	(SD)	N	M	(SD)	N	M	(SD)
CES-D	T00	264	25.85	(7.94)	249	26.29	(7.66)	266	25.15	(6.96)
	T0	264	27.36	(8.19)	249	27.36	(7.37)	266	26.17	(6.97)
	T1	212	24.51	(9.59)	194	25.30	(10.03)	223	25.17	(8.74)
	T2	202	25.25	(9.79)	184	25.54	(10.09)	212	24.72	(8.86)
	T3	197	24.00	(10.36)	179	25.51	(10.03)	182	22.87	(8.86)
PHQ-9	T00	264	10.53	(3.79)	249	10.52	(3.97)	266	10.39	(3.90)
	T0	264	11.06	(4.57)	249	11.27	(4.43)	266	10.81	(4.09)
	T1	212	9.75	(4.95)	194	9.95	(5.45)	223	10.42	(5.04)
	T2	202	10.46	(5.49)	184	10.27	(5.64)	212	10.20	(5.11)
	T3	197	9.89	(5.74)	179	10.35	(5.84)	182	9.06	(4.47)
GAD-7	T0	264	8.04	(4.42)	249	8.38	(4.74)	266	7.66	(4.13)
	T1	212	6.83	(4.69)	194	7.56	(4.96)	223	7.18	(4.54)
	T2	202	7.65	(5.21)	184	7.94	(5.34)	212	7.30	(4.69)
	T3	197	7.08	(4.98)	179	8.01	(5.23)	182	6.96	(4.46)

*iCBT* internet-based cognitive behavioral therapy, *sEFM* simplified emotion-focused mindfulness. T00 = initial screening (only for CES-D and PHQ-9), T0 = baseline, T1 = postintervention, T2 = six weeks after T1, T3 = six weeks after T2. *CES-D* the Center for Epidemiological Studies Depression scale, *PHQ-9* the Patient Health Questionnaire-9, *GAD-7* the Generalized Anxiety Disorder-7

**Table 5:** Linear mixed model analyses limited to those who met inclusion criteria at baseline (CES-D ≥ 16 and PHQ-9 ≥ 5)

		Predicted means (95% CI)			Intervention effect (95% CI), *p* value		
	Time	iCBT	sEFM	Waiting list	iCBT – waiting list	sEFM – waiting list	iCBT – sEFM
CES-D	T0	27.16 (26.39, 27.93)	27.16 (26.37, 27.95)	26.92 (26.15, 27.69)			
	T1	24.08 (23.23, 24.93)	24.70 (23.81, 25.59)	25.94 (25.10, 26.77)	**−2.09 (−3.54, −0.65); <0.01**	**−**1.47 (**−**2.95,0.00); 0.05	**−**0.62 (**−**2.11,0.87); 0.42
	T2	24.63 (23.76, 25.50)	24.95 (24.04, 25.86)	25.49 (24.63, 26.34)	**−**1.10 (**−**2.56,0.37); 0.14	**−**0.77 (**−**2.27,0.73); 0.31	**−**0.32 (**−**1.84,1.19); 0.68
	T3	23.39 (22.44, 24.34)	25.22 (24.23, 26.22)				**−1.83 (−3.39, −0.28); 0.02**
PHQ-9	T0	11.04 (10.61, 11.48)	11.09 (10.64, 11.54)	10.99 (10.56, 11.42)			
	T1	9.65 (9.16, 10.13)	9.70 (9.19, 10.2)	10.72 (10.25, 11.19)	**−1.13 (−1.97, −0.29); 0.01**	**−1.12 (−1.98, −0.27); 0.01**	0.00 (**−**0.87,0.86); 0.99
	T2	10.33 (9.84, 10.82)	10.05 (9.53, 10.56)	10.57 (10.08, 11.05)	**−**0.29 (**−**1.14,0.56); 0.50	**−**0.62 (**−**1.49,0.25); 0.16	0.33 (**−**0.54,1.21); 0.46
	T3	9.72 (9.19, 10.25)	10.20 (9.64, 10.76)				**−**0.43 (**−**1.32,0.47); 0.35
GAD-7	T0	8.05 (7.66, 8.44)	8.12 (7.72, 8.53)	7.96 (7.57, 8.35)			
	T1	6.74 (6.31, 7.18)	7.18 (6.73, 7.64)	7.47 (7.05, 7.90)	**−0.82 (−1.57, −0.06); 0.03**	**−**0.45 (**−**1.22,0.32); 0.25	**−**0.37 (**−**1.14,0.41); 0.36
	T2	7.61 (7.16, 8.05)	7.66 (7.19, 8.13)	7.63 (7.19, 8.06)	**−**0.11 (**−**0.88,0.66); 0.78	**−**0.13 (**−**0.91,0.65); 0.75	0.02 (**−**0.77,0.81); 0.96
	T3	7.02 (6.55, 7.50)	7.90 (7.40, 8.40)				**−**0.79 (**−**1.58,0.00); 0.05

*iCBT* internet-based cognitive behavioral therapy, *sEFM* simplified emotion-focused mindfulness. *CI* confidence interval. T0 = baseline, T1 = postintervention, T2 = six weeks after T1, T3 = six weeks after T2. *CES-D* the Center for Epidemiological Studies Depression scale, *PHQ-9* the Patient Health Questionnaire-9, *GAD-7* the Generalized Anxiety Disorder-7. For T0 through T2, predicted means (95% CI) from mixed model with outcome measure at baseline, time (T0, T1, T2), treatment (iCBT, sEFM, Waiting list), and interaction between time and treatment as fixed effects. For T3, predicted means (95% CI) from mixed model with outcome measure at baseline, time (T0, T1, T2, T3), treatment (iCBT, sEFM, delayed iCBT, delayed sEFM), and interaction between time and treatment as fixed effects. Fixed effect of time × treatment interaction as an indicator of intervention effect. Values in bold are significant at 5%

#### Effects of the iCBT and sEFM compared with those of the waiting list

Regarding the CES-D, which is the primary outcome measure, when limiting to those who met the inclusion criteria at baseline assessment, there was a significant difference in favor of the iCBT (−2.09, 95% CI: −3.54 to −0.65, *p* < 0.01) and an almost significant difference in favor of the sEFM arm (−1.47, 95% CI: −2.95 to 0.00, *p* = 0.05) as compared with the waiting list arm at T1, respectively.

Regarding the PHQ-9 as the secondary outcome, when limited to those who met the inclusion criteria at baseline assessment, there were significant differences between iCBT and the waiting list in favor of iCBT (−1.13, 95% CI: −1.97 to −0.29, *p* = 0.01) and between sEFM and the waiting list at T1 in favor of sEFM (−1.12, 95% CI: −1.98 to −0.27, *p* = 0.01), respectively.

Regarding the GAD-7, when limited to those who met the inclusion criteria at baseline assessment, there was a significant difference in favor of the iCBT (−0.82, 95% CI: −1.57 to −0.06, *p* = 0.03) as compared with the waiting list arm at T1 but not for the sEFM.

#### Comparison of the iCBT and sEFM

Regarding comparison of the iCBT and sEFM, there were significant difference at T3 (six weeks after T2) in favor of iCBT on the CES-D (−1.83, 95% CI: −3.39 to −0.28, *p* = 0.02) and marginally significant difference in favor of iCBT on the GAD-7 (−0.79, 95% CI: −1.58 to 0.00, *p* = 0.05), respectively.

### Analyses of subgroups for non-, mild, and moderately and more severe depressed people

The means and standard deviations for outcome measures on all the participants are shown in Table 6, which reflect the severity of depression at baseline (T0). The results of the analyses are shown in Table 7. First, we compared each intervention group with the waiting list group. In the nondepressed group at baseline, based on the CES-D (CES-D < 16), there were significant differences between iCBT and waiting list at T1 and between sEFM and waiting list at T2 in favor of the waiting list arm (3.39, 95% CI: 0.24 to 6.54, *p* = 0.03; 4.53, 95% CI: 1.42 to 7.64, *p* < 0.01, respectively). There were no significant differences between any group and at any time point in the mildly depressed group (16 ≤ CES-D < 26). In the moderately and more severely depressed group (CES-D ≥ 26), there were significant differences between iCBT and waiting list at T1 in favor of iCBT (−2.98, 95% CI: −5.28 to −0.68, *p* < 0.01). In the nondepressed group (PHQ-9 < 5) and the mildly depressed group (5 ≤ PHQ-9 < 10) at baseline, based on the PHQ-9, there were no significant differences between any group and at any time point. For moderately and more severely depressed participants (PHQ-9 ≥ 10), there were significant differences between iCBT and waiting list in favor of iCBT (−1.31, 95% CI: −2.51 to −0.11, *p* = 0.03) and between sEFM and waiting list at T1 in favor of sEFM (−1.34, 95% CI: −2.57 to −0.11, *p* = 0.03), respectively.

**Table 6:** Means and standard deviations at each time point depending on severity of depression at baseline (T0)

		iCBT			sEFM			Waiting list		
	Time	N	M	(SD)	N	M	(SD)	N	M	(SD)
CES-D	T00	45	18.98	(3.50)	48	19.25	(3.90)	45	19.02	(2.93)
CES-D < 16	T0	45	12.47	(2.58)	48	12.60	(1.97)	45	12.22	(2.85)
	T1	33	18.76	(3.56)	40	16.38	(6.67)	39	14.64	(6.64)
	T2	29	17.07	(3.31)	38	18.21	(7.45)	35	13.37	(5.53)
	T3	28	16.32	(6.48)	34	15.85	(6.96)	33	12.91	(6.51)
16 ≤ CES-D < 26	T00	148	21.94	(4.95)	141	22.16	(4.83)	161	22.01	(4.64)
	T0	148	20.65	(2.81)	141	20.60	(3.07)	161	21.04	(2.76)
	T1	114	20.30	(6.80)	102	20.53	(7.96)	134	21.09	(6.63)
	T2	107	20.67	(7.54)	95	20.82	(8.01)	130	21.52	(6.55)
	T3	103	19.80	(7.85)	93	20.83	(7.85)	110	19.53	(7.04)
26 ≤ CES-D	T00	133	29.85	(8.25)	134	29.60	(7.99)	119	28.97	(7.39)
	T0	133	33.75	(6.58)	134	32.65	(5.71)	119	32.43	(5.32)
	T1	107	28.56	(10.18)	110	28.66	(10.10)	102	29.74	(8.74)
	T2	104	29.55	(9.71)	105	28.93	(10.25)	95	28.57	(9.76)
	T3	102	28.02	(10.78)	99	28.88	(10.37)	82	26.68	(9.56)
PHQ-9	T00	38	6.97	(1.95)	49	6.67	(1.80)	35	7.26	(1.96)
PHQ-9 < 5	T0	38	2.45	(1.33)	49	2.78	(1.16)	35	2.97	(1.15)
	T1	22	6.00	(3.56)	34	4.06	(2.85)	32	4.84	(2.83)
	T2	21	5.43	(3.31)	32	5.47	(5.47)	30	4.60	(3.62)
	T3	20	5.75	(4.35)	27	4.22	(4.22)	26	5.04	(4.44)
	T00	135	8.06	(2.25)	132	8.13	(2.59)	141	8.24	(2.36)
5 ≤ PHQ-9 < 10	T0	135	7.04	(1.37)	132	7.21	(1.39)	141	7.16	(1.36)
	T1	104	7.38	(3.82)	104	6.88	(4.28)	123	7.73	(3.78)
	T2	99	7.73	(4.20)	97	7.19	(4.43)	117	7.38	(3.42)
	T3	94	6.85	(4.34)	93	7.94	(4.30)	105	6.96	(3.84)
10 ≤ PHQ-9	T00	153	12.20	(3.77)	142	12.19	(4.03)	149	12.04	(4.05)
	T0	153	13.95	(3.88)	142	14.20	(3.66)	149	13.60	(3.30)
	T1	128	11.42	(4.89)	114	11.72	(5.46)	120	12.48	(5.11)
	T2	120	12.42	(5.50)	109	12.17	(5.62)	113	12.46	(5.22)
	T3	119	11.86	(5.58)	106	11.82	(6.28)	94	10.73	(4.40)

*iCBT* internet-based cognitive behavioral therapy, *sEFM* simplified emotion-focused mindfulness. T00 = initial screening, T0 = baseline, T1 = postintervention, T2 = six weeks after T1, T3 = six weeks after T2. *CES-D* the Center for Epidemiological Studies Depression scale, *PHQ-9* the Patient Health Questionnaire-9

**Table 7:** Linear mixed model analyses depending on severity of depression at baseline

		Predicted means (95% CI)			Intervention effect (95% CI), *p* value		
	Time	iCBT	sEFM	Waiting list	iCBT – waiting list	sEFM – waiting list	iCBT – sEFM
CES-D	T0	12.51 (10.84, 14.18)	12.55 (10.93, 14.17)	12.45 (10.77, 14.12)			
CES-D < 16	T1	18.38 (16.44, 20.32)	16.33 (14.57, 18.10)	14.92 (13.13, 16.71)	**3.39 (0.24,6.54); 0.03**	1.31 (−1.71,4.34); 0.40	2.08 (−1.04,5.20); 0.19
	T2	16.64 (14.58, 18.70)	18.13 (16.33, 19.93)	13.50 (11.62, 15.38)	3.07 (−0.21,6.35); 0.07	**4.53 (1.42,7.64); <0.01**	−1.46 (−4.68,1.76); 0.37
	T3	16.08 (13.95, 18.20)	15.77 (13.83, 17.70)				0.35 (−2.90,3.60); 0.83
16 ≤ CES-D < 26	T0	20.82 (19.96, 21.68)	20.81 (19.93, 21.70)	20.88 (20.06, 21.71)			
	T1	20.28 (19.31, 21.26)	20.51 (19.48, 21.54)	21.09 (20.19, 21.99)	−0.74 (−2.33,0.84); 0.36	−0.51 (−2.14,1.11); 0.54	−0.23 (−1.91,1.44); 0.79
	T2	20.55 (19.55, 21.56)	20.95 (19.88, 22.01)	21.44 (20.52, 22.35)	−0.82 (−2.43,0.79); 0.32	–0.42 (−2.07,1.24); 0.62	−0.40 (−2.12,1.31); 0.64
	T3	19.64 (18.54, 20.75)	20.86 (19.71, 22.02)				−1.23 (−3.02,0.56); 0.18
26 ≤ CES-D	T0	33.18 (31.98, 34.38)	32.95 (31.76, 34.15)	32.91 (31.64, 34.18)			
	T1	27.59 (26.26, 28.93)	28.96 (27.65, 30.28)	30.31 (28.94, 31.67)	**−2.98 (−5.28,−0.68); 0.01**	−1.39 (−3.67,0.90); 0.23	−1.59 (−3.84,0.65); 0.16
	T2	28.60 (27.25, 29.95)	29.15 (27.81, 30.50)	29.06 (27.65, 30.47)	−0.72 (−3.06,1.61); 0.54	0.05 (−2.28,2.38); 0.96	−0.78 (−3.05,1.50); 0.50
	T3	27.18 (25.71, 28.65)	29.23 (27.74, 30.71)				−2.32 (−4.66,0.01); 0.05
PHQ-9	T0	2.59 (1.77, 3.42)	2.77 (2.05, 3.50)	2.88 (2.02, 3.74)			
PHQ-9 < 5	T1	6.04 (4.97, 7.11)	4.05 (3.19, 4.91)	4.77 (3.88, 5.67)	1.55 (−0.11,3.21); 0.07	−0.61 (−2.12,0.90); 0.43	**2.16 (0.56,3.77); 0.01**
	T2	5.52 (4.43, 6.61)	5.53 (4.65, 6.42)	4.52 (3.59, 5.44)	1.29 (−0.40,2.98); 0.13	1.12 (−0.41,2.66); 0.15	0.17 (−1.47,1.80); 0.84
	T3	5.87 (4.60, 7.13)	4.23 (3.15, 5.31)				1.86 (−0.00,3.72); 0.05
5 ≤ PHQ-9 < 10	T0	7.11 (6.61, 7.61)	7.13 (6.62, 7.64)	7.12 (6.63, 7.62)			
	T1	7.45 (6.88, 8.03)	6.90 (6.33, 7.47)	7.70 (7.18, 8.23)	−0.23 (−1.18,0.71); 0.63	−0.81 (−1.76,0.14); 0.09	0.58 (−0.40,1.55); 0.25
	T2	7.77 (7.18, 8.35)	7.23 (6.64, 7.82)	7.38 (6.84, 7.91)	0.41 (−0.56,1.37); 0.41	−0.15 (−1.12,0.82); 0.76	0.55 (−0.44,1.55); 0.28
	T3	6.91 (6.27, 7.55)	7.95 (7.31, 8.59)				−1.02 (−2.06,0.02); 0.06
10 ≤ PHQ-9	T0	13.92 (13.30, 14.54)	13.99 (13.34, 14.63)	13.83 (13.20, 14.46)			
	T1	11.45 (10.78, 12.13)	11.49 (10.77, 12.20)	12.67 (11.97, 13.37)	**−1.31 (−2.51,−0.11); 0.03**	**−1.34 (−2.57,−0.11); 0.03**	0.03 (−1.19,1.25); 0.96
	T2	12.33 (11.63, 13.02)	12.00 (11.27, 12.73)	12.70 (11.98, 13. · 42)	−0.46 (−1.69,0.76); 0.46	−0.86 (−2.11,0.39); 0.18	0.40 (−0.84,1.63); 0.53
	T3	11.78 (11.03, 12.52)	11.75 (10.96, 12.53)				0.10 (−1.13,1.34); 0.87

*iCBT* internet-based cognitive behavioral therapy, *sEFM* simplified emotion-focused mindfulness. *CI* confidence interval. T0 = baseline, T1 = postintervention, T2 = six weeks after T1, T3 = six weeks after T2. *CES-D* the Center for Epidemiological Studies Depression scale, *PHQ-9* the Patient Health Questionnaire-9. For T0 through T2, predicted means (95% CI) from mixed model with outcome measure at baseline, time (T0, T1, T2), treatment (iCBT, sEFM, Waiting list), and interaction between time and treatment as fixed effects. For T3, predicted means (95% CI) from mixed model with outcome measure at baseline, time (T0, T1, T2, T3), treatment (iCBT, sEFM, delayed iCBT, delayed sEFM), and interaction between time and treatment as fixed effects. Fixed effect of time × treatment interaction as an indicator of intervention effect. Values in bold are significant at 5%

Comparisons of the iCBT arm and the sEFM arm showed that there were no significant differences between the two groups in both the CES-D and PHQ-9, except that there was a significant difference in favor of sEFM at T1 in nondepressed participants in the PHQ-9 (2.16, 95% CI:0.56 to 3.77, *p* = 0.01). The difference between the moderately and more severely depressed groups in the CES-D at T3 was close to significant in favor of iCBT (−2.32, 95% CI: −4.66 to 0.01, *p* = 0.05).

### Comparing delayed iCBT arm and delayed sEFM arm

Those who belonged to the waiting list arm were randomly assigned to the delayed iCBT arm and the delayed sEFM arm after T2 assessment, and they performed iCBT or sEFM. There were no significant differences between the two groups at any time point in all outcome measures. (Additional file 3: Table S1; Additional file 4: Table S2).

## Discussion

The present study examined whether or not simplified iCBT and sEFM exercises reduce depressive symptoms compared with the effects of a waiting list control group. The results showed that there were no significant differences between the intervention groups and the control group in the primary outcome measure (CES-D) at postintervention (T1), although the difference between the iCBT arm and the control group was almost significant (*p* = 0.05) in favor of iCBT. In the PHQ-9, there were significant differences in depressive symptoms in favor of the sEFM group as compared with the control group at postintervention (T1). The results in favor of interventions were not maintained at the 6-week follow-up (T2). Although there were no significant differences between iCBT and sEFM at postintervention (T1) and the six-week follow-up (T2), there was a significant difference between them in the CES-D and GAD-7 in favor of the iCBT arm at the 12-week follow-up (T3). When limited to those who met the inclusion criteria at baseline assessment (CES-D ≥ 16 and PHQ-9 ≥ 5), there were significant or almost significant differences between the intervention groups and the control group in both CES-D and PHQ-9 at postintervention (T1). In the GAD-7, there was a significant difference in favor of the iCBT group as compared with the control group at postintervention (T1).

Overall, the present study showed that both iCBT and sEFM were effective in reducing depressive symptoms in moderately and more severely depressed participants, but the effect was small and temporary. No differences of effects between iCBT and sEFM were found except that symptoms of depression and anxiety may be fewer in favor of iCBT in the long run (12 weeks after the completion of the exercise period).

Considering the small and temporary effects of both interventions, the improvement of treatment designs on the basis of the lessons learned from the present study is required. Regarding iCBT, plausible explanations of the limited effects are that the exercises in the present study may have been too simplistic and that sufficient instruction was not given to the participants. The exercises were initiated without sufficient explanation of the mechanism and concepts of CBT, such as the cognitive triad, cognitive distortion, and maladaptive behavior [31]. It may be desirable to explain such mechanisms of iCBT to the participants in the initial stages of the intervention period using YouTube videos and/or a printable PDF brochure, which are compatible with online intervention. Another point to consider in iCBT is that 5-min sessions for 5 weeks may be too short. Typical one-to-one CBT sessions are an hour or more with a therapist once a week for 12 to 20 weeks plus homework [31]. It may be better to extend the duration of simplified iCBT, for example, to 10 min for 10 weeks, although the possibility of increased drop-out rate should be considered.

The abovementioned points on iCBT may also be applicable to sEFM; sufficient explanation and longer duration may improve its effects. In addition to this, one possible problem for sEFM in this study is that the participants were instructed to come up with recent experiences that were slightly uncomfortable. There were comments from several participants that being reminded of upsetting events was painful. Although there is sufficient reason to believe that encouraging acceptance of emotions rather than challenging them leads to the alleviation of negative emotions [9], going so far as activating uncomfortable memories may not be a good treatment strategy, especially for a self-help setting. To the authors’ knowledge, this point has not been empirically tested, and further exploration is expected.

Several explanations can be explored on why there were no significant differences between iCBT and sEFM. One possibility is that despite the apparent differences between the two modalities, their actual mechanisms may neurophysiologically and psychologically overlap. A recent functional magnetic resonance imaging study found that “both acceptance and reappraisal showed similar patterns of prefrontal cortex activation in both individuals remitted from depression as well as never-depressed controls, with a few notable exceptions” ([19], p. 1192). Emotional acceptance, by giving nonjudgmental attention to negative emotions, may lead to a more objective interpretation of events and the spontaneous reappraisal (cognitive restructuring) of the emotion arousing events [9, 32].

Another possible explanation for the lack of significant differences between the two modalities is that the participants may have varied in the extent to which the strategies they used aligned with fidelity to the treatment instructions. There was no comprehensive guidance from the authors and no two-way communication between the participants and authors, which may have caused varied fidelity and different interpretations of treatment instructions. The variety of fidelity may have been augmented by the study setting in which the participants had not been proactively looking for treatment for their depressive symptoms by themselves in the study; thus, they were rather reactive and able to participate simply by responding to the invitations.

Although we have so far focused on the improvements of iCBT and sEFM per se, combining several modalities may also be useful. One potential approach to investigate in this direction is combining reappraisal and acceptance in one exercise. Shallcross and her colleagues suggested that by engaging emotional acceptance prior to performing cognitive reappraisal, the effectiveness of cognitive reappraisal may be strengthened [9]. Based on this idea, one possible improvement to the treatment strategy is to combine the two modalities and perform a 5-minute iCBT after giving a few minutes for feeling the negative emotions caused by activating events, thereby making it easier to come up with less emotionally charged interpretations of events.

Another potential investigation approach would be combining the exercises used in the present study with gratitude exercises based on positive psychology [33]. In contrast with focusing on negative events, emotions, and thoughts by simplified iCBT and sEFM, positive events and emotions are viewed in the so-called three good things exercise in which participants are encouraged to write three good things at night before going to bed [33]. A variety of online depression interventions may be necessary for people with subclinical depression to continue without losing interest. If people can take 5 more minutes to talk to someone about their cognitive changes and emotional changes after our simple and free exercises, the positive effects of depressive symptoms may be increased. To develop simple and free online anti-depression exercises, further studies should be conducted.

The present study has several limitations. First, the participants were selected using cutoff points drawn from depression severity scales (CES-D and PHQ-9). We did not use a structured diagnostic interview schedule for a diagnosis of depression as the inclusion criteria. Second, because of the time lag between the initial screening and baseline assessment, those who were not depressed as reflected in the CES-D and PHQ-9 scores at baseline were included in the study. This made the interpretation of the present study difficult. Third, the participants were recruited from people registered on the list of a market research company to be monitors for the company’s market research. Thus, we cannot be sure whether the interventions used in the present study would work in a clinical setting. We also cannot be certain whether the interventions would be effective for a broader range of people, considering that the rate of participation of college graduate students was high in the present study. Fourth, the participants in this study exhibited a wide range of severity of depression. Fifth, the follow-up time to compare the intervention groups with the control group was inadequate; the follow-up time was 6 weeks after the end of the interventions and those people belonging to the control group began delayed interventions after that. We took this approach as it seemed unethical to make the waiting list control group hold back for such a long time. However, for the purposes of the research, this setting made comparisons more difficult. Sixth, the number of participants with nondepression at baseline was low and it may be inappropriate to generalize our study’s findings to nondepressed people. Further research for nondepressed people is anticipated. Seventh, as the present study was a comparison between the treatment groups and waiting list group without intervention, it is possible that the temporary effect in the intervention groups was a placebo effect. In the future, further studies to compare iCBT and/or sEFM with a psychological placebo group are needed.

## Conclusions

Searching for a way to devise simple and less costly methods for reducing depressive symptoms, we examined the effects of a free, simplified, 5-minute iCBT program, comparing it with a sEFM exercise and with a waiting list control group. During postintervention assessment, no significant differences were found between the intervention arms and the control group in the primary outcome measure (CES-D), although the difference between the iCBT arm and the control group was close to significance in favor of iCBT. There was a significant difference in secondary outcome measure (PHQ-9) in favor of the sEFM group compared with the control group. This study on 5-minute iCBT and sEFM, which need substantial improvements to enhance and maintain their effects, may be useful for guiding simple and free online depression intervention development.

## Additional files

Additional file 1: Five-minute Internet-based Cognitive Behavioral Therapy Exercise (English translation, original in Japanese). (DOCX 39 kb)
Additional file 2: Simplified Emotion-focused Mindfulness Exercise (English translation, original in Japanese). (DOCX 37 kb)
Additional file 3: Table S1. Means and Standard Deviations at Each Time Point (Delayed iCBT arm and Delayed sEFM arm). (DOCX 38 kb)
Additional file 4: Table S2. Linear Mixed Model Analyses on Delayed iCBT Arm and Delayed sEFM Arm. (DOCX 38 kb)

## Abbreviations

CES-D: Center for epidemiological studies depression scale
GAD-7: Generalized anxiety disorder-7 scale
iCBT: Internet-based cognitive behavioral therapy
PHQ-9: Patient health questionnaire-9 scale
RCT: Randomized controlled trial
RIETI: Research institute of economy trade and industry
sEFM: Simplified emotion-focused mindfulness
