# Supplementary material for: Effects of five-minute internet-based cognitive behavioral therapy and simplified emotion-focused mindfulness on depressive symptoms: a randomized controlled trial
Source: BMC Psychiatry. 2017 Mar 4;17:85. doi: 10.1186/s12888-017-1248-8 (PMC5336676; doi:10.1186/s12888-017-1248-8)
Supplement: Additional file 1: — Five-minute Internet-based Cognitive Behavioral Therapy Exercise (English translation, original in Japanese). (DOCX 39 kb) [file 12888_2017_1248_MOESM1_ESM.docx]

Additional file 1

Five-minute Internet-based Cognitive Behavioral Therapy Exercise

(English translation, original in Japanese)

1. What makes you feel annoyed or stressed? Include “I think that” before your response.

Your sentence should comprise approximately 50 letters.

I think that .

2. To what extent do you think the above-mentioned thought is true? Rate it between 0 and 100.

3. To what extent does the above-mentioned thought cause you to feel suffering? Rate it between 0 and 100.

4. Change the above-mentioned thought into its opposite (Negative 　　　 Positive).

I think that .

5. Write down two specific examples or types of evidence to support the new thought.

1)

2)

6. Think again about the thought you wrote down in 1. To what extent do you think your original thought is true? Rate it between 0 and 100.

7. To what extent does your original thought cause you to feel suffering? Rate it between 0 and 100.

8. Your evaluation of your original thought changed from to .

9. Your evaluation of your suffering changed from to .
