# Supplementary material for: Effects of five-minute internet-based cognitive behavioral therapy and simplified emotion-focused mindfulness on depressive symptoms: a randomized controlled trial
Source: BMC Psychiatry. 2017 Mar 4;17:85. doi: 10.1186/s12888-017-1248-8 (PMC5336676; doi:10.1186/s12888-017-1248-8)
Supplement: Additional file 2: — Simplified Emotion-focused Mindfulness Exercise (English translation, original in Japanese). (DOCX 37 kb) [file 12888_2017_1248_MOESM2_ESM.docx]

Additional file 2

Simplified Emotion-focused Mindfulness Exercise

(English translation, original in Japanese)

Do you acknowledge your emotions and feel them fully? We may try to avoid feeling our emotions and suppress them deep inside without noticing we are doing it. We put lids on uncomfortable feelings such as depression, anger, sadness, and anxiety. Although suppressing uncomfortable emotions may seem to make you feel better at the time, it does not work. If you resist your feelings, they will persist. So let’s do an exercise to acknowledge your feelings and feel them fully.

Find a place where you can be alone if possible. First, feel your feelings. What are you feeling now? Are you feeling uncomfortable feelings such as anger, sadness, and anxiety? If not, try to feel them by, for example, reminding yourself of a slightly uncomfortable experience you had recently. When you feel a negative feeling, acknowledge it. Be with the feeling. Just feel it. Savor it. Set aside the thought that you should not feel such a feeling. Just feel the feeling without denying it. Give yourself permission to feel whatever feeling you feel.

If you feel angry, just acknowledge the anger and feel it. You do not have to hide anger even toward your loved ones from yourself. Just feel the anger toward anyone without denying it. However, don’t vent your anger at the person. Be alone if possible, and just feel the anger. If you feel like crying due to sadness, just cry. You don’t have to refrain from crying. Just cry in a place where no one is around. If you feel anxiety or fear and think that you should not feel these feelings, set aside the thought and just be with the anxiety or fear. Acknowledge the anxiety and just feel it.

If it is difficult to be with emotions (just feel the emotions), pay attention to your body sensations. Does your body react when you feel some emotion? Do you feel tightness in your stomach, chest, or neck? Or do you have a racing pulse? Or does your body shake? If you have such bodily sensations, pay attention to them and be with them.

If you begin to think about something when you are doing this exercise, bring your attention back to the feeling. This often happens. If you begin to think, just be with the feeling again. If you feel nothing, be with the sensation of nothing. Treat the sensation of no feeling as a kind of feeling.

It is suggested that the exercise should last 10 minutes. But it can be longer and it can be less. Follow your intuition. If you experience upsetting events, take time later to be alone and do the exercise by looking back at the events.

Don’t do this exercise when you are drunk. If you plan to drink, do this exercise before drinking.

The instructions for this exercise are the same for every day. You will experience the effect of the exercise as you continue to do it. Try to do the exercise as best you can without putting too much of a burden on yourself.
