# Supplementary material for: Effects of five-minute internet-based cognitive behavioral therapy and simplified emotion-focused mindfulness on depressive symptoms: a randomized controlled trial
Source: BMC Psychiatry. 2017 Mar 4;17:85. doi: 10.1186/s12888-017-1248-8 (PMC5336676; doi:10.1186/s12888-017-1248-8)
Supplement: Additional file 3: Table S1. — Means and Standard Deviations at Each Time Point (Delayed iCBT arm and Delayed sEFM arm). (DOCX 38 kb) [file 12888_2017_1248_MOESM3_ESM.docx]

Additional file 3

| Table S1. Means and Standard Deviations at Each Time Point  (Delayed iCBT arm and Delayed sEFM arm) | | | | | | | |
| --- | --- | --- | --- | --- | --- | --- | --- |
|  |  | Delayed iCBT | | | Delayed sEFM | | |
|  | Time | N | M | (SD) | N | M | (SD) |
|  | T00 | 162 | 24.59 | (7.25) | 163 | 23.70 | (6.30) |
| CES-D | T0 | 162 | 23.77 | (8.44) | 163 | 24.20 | (7.67) |
|  | T1 | 137 | 22.93 | (9.47) | 138 | 23.83 | (8.87) |
|  | T2 | 128 | 22.63 | (9.97) | 132 | 23.36 | (8.45) |
|  | T3 | 110 | 20.98 | (8.74) | 115 | 21.34 | (9.77) |
|  | T00 | 162 | 9.77 | (3.86) | 163 | 9.99 | (3.73) |
| PHQ-9 | T0 | 162 | 9.68 | (4.79) | 163 | 9.65 | (4.29) |
|  | T1 | 137 | 9.35 | (5.37) | 138 | 9.58 | (4.91) |
|  | T2 | 128 | 9.07 | (5.46) | 132 | 9.45 | (4.96) |
|  | T3 | 110 | 8.06 | (4.44) | 115 | 8.56 | (4.85) |
| GAD-7 | T0 | 162 | 6.77 | (4.47) | 163 | 6.98 | (4.11) |
|  | T1 | 137 | 6.53 | (4.73) | 138 | 6.67 | (4.33) |
|  | T2 | 128 | 6.31 | (4.68) | 132 | 6.90 | (4.57) |
|  | T3 | 110 | 6.10 | (4.46) | 115 | 6.49 | (4.58) |
| *Note.* iCBT = internet-based cognitive behavioral therapy; sEFM = simplified emotion-focused mindfulness. T00 = initial screening (only for CES-D and PHQ-9), T0 = baseline, T1 = postintervention, T2 = six weeks after T1, T3 = six weeks after T2. CES-D = the Center for Epidemiological Studies Depression scale, PHQ-9 = the Patient Health Questionnaire-9, GAD-7 = the Generalized Anxiety Disorder-7. Delayed iCBT arm and delayed sEFM arm were the waiting list control until the end of T2. After the evaluation at T2, they started their respective exercise. Assessment at T3 is their postintervention assessment. | | | | | | | |
