# Supplementary material for: Effects of five-minute internet-based cognitive behavioral therapy and simplified emotion-focused mindfulness on depressive symptoms: a randomized controlled trial
Source: BMC Psychiatry. 2017 Mar 4;17:85. doi: 10.1186/s12888-017-1248-8 (PMC5336676; doi:10.1186/s12888-017-1248-8)
Supplement: Additional file 4: Table S2. — Linear Mixed Model Analyses on Delayed iCBT Arm and Delayed sEFM Arm. (DOCX 38 kb) [file 12888_2017_1248_MOESM4_ESM.docx]

Additional file 4

| Table S2. Linear Mixed Model Analyses on Delayed iCBT Arm and Delayed sEFM Arm | | | | |
| --- | --- | --- | --- | --- |
|  |  | Predicted means (95% CI) | | Intervention effect (95% CI), *p* value |
|  | Time | Delayed iCBT | Delayed sEFM | Delayed iCBT – Delayed sEFM |
| CES-D | T0 | 23.88 (22.96, 24.81) | 23.97 (23.04, 24.89) |  |
|  | T1 | 23.24 (22.24, 24.24) | 23.57 (22.57, 24.56) | −0.24 (−1.90,1.41); 0.77 |
|  | T2 | 22.84 (21.81, 23.88) | 22.97 (21.96, 23.99) | −0.05 (−1.73,1.64); 0.96 |
|  | T3 | 21.50 (20.40, 22.60) | 20.91 (19.83, 21.99) | 0.68 (−1.09,2.44); 0.45 |
| PHQ-9 | T0 | 9.53 (8.99, 10.06) | 9.52 (8.99, 10.05) |  |
|  | T1 | 9.40 (8.83, 9.97) | 9.52 (8.95, 10.10) | −0.13 (−1.08,0.82); 0.79 |
|  | T2 | 9.18 (8.58, 9.77) | 9.39 (8.80, 9.97) | −0.22 (−1.19,0.75); 0.66 |
|  | T3 | 8.35 (7.72, 8.98) | 8.56 (7.94, 9.18) | −0.21 (−1.23,0.80); 0.68 |
| GAD-7 | T0 | 6.84 (6.38, 7.30) | 6.88 (6.43, 7.34) |  |
|  | T1 | 6.65 (6.16, 7.14) | 6.55 (6.06, 7.05) | 0.14 (−0.69,0.97); 0.74 |
|  | T2 | 6.51 (6.00, 7.02) | 6.77 (6.26, 7.27) | −0.21 (−1.05,0.63); 0.62 |
|  | T3 | 6.38 (5.84, 6.92) | 6.09 (5.55, 6.62) | 0.34 (−0.55,1.22); 0.45 |
| *Note.* iCBT = internet-based cognitive behavioral therapy; sEFM = simplified emotion-focused mindfulness. T0 = baseline, T1 = postintervention, T2 = six weeks after T1, T3 = six weeks after T2. CES-D = the Center for Epidemiological Studies Depression scale, PHQ-9 = the Patient Health Questionnaire-9, GAD-7 = the Generalized Anxiety Disorder-7. Predicted means (95% CI) from mixed model with outcome measure at baseline, time (T0, T1, T2, T3), treatment (delayed iCBT, delayed sEFM), and interaction between time and treatment as fixed effects. Fixed effect of time × treatment interaction as an indicator of intervention effect. Delayed iCBT arm and delayed sEFM arm were the waiting list control until the end of T2. After the evaluation at T2, they started their respective exercise. Assessment at T3 is their postintervention assessment. | | | | |
